# The compass, crow’s nest and ship of medicine in the sea of uncertainties

**DOI:** 10.1590/S1516-31802009000100001

**Published:** 2009-05-15

**Authors:** Álvaro Nagib Atallah

**Affiliations:** 1 Physician. Full professor and head of the Discipline of Emergency Medicine and Evidence-Based Medicine of Universidade Federal de São Paulo - Escola Paulista de Medicina (Unifesp-EPM). Director of the Brazilian Cochrane Center and Scientific Director of Associação Paulista de Medicina (APM). E-mail: atallahmbe@uol.com.br.

Clinical trials have progressively been providing fundamental support for reducing the uncertainties in decision-making in Medicine. Their benefits can be seen historically and are indisputable.

Since Lind’s celebrated clinical trial in 1753, which demonstrated that scurvy could be treated with citric fruits, this simple and objective study design has improved and continues to improve healthcare as a whole. It suffices to say that before Lind’s comparative study, around 50% of the crews of ships involved in long voyages died of scurvy. In 1807-1808, Dom João VI arrived in Brazil accompanied by an entourage of around 16,000 people, in heavily laden caravels, some with about 1,200 people, as narrated in the magnificently researched and reported book “1808” by Laurentino Gomes. There were no deaths and, if scurvy occurred, it was not fatal despite all the discomfort and lack of hygiene that was commonplace at that time. It can be said in passing that, even though Lind’s study was published in 1753, the British Navy only implemented treatment (and prevention) of scurvy 50 years later. On the other hand, the Portuguese received the benefit from the scurvy prevention routine that the American Navy had started to disseminate two years before the voyage of the Portuguese Royal Court.^1^

When should a clinical trial be conducted and when should it be used? We would say that there is ethical and scientific space for conducting a clinical trial when there is uncertainty regarding one or more interventions. Recently, a study by Dossett et al. called “Cost-effectiveness of routine radiographs after emergent open cavity operations” was published in the journal Surgery. This paper attempted to reduce the uncertainty regarding the effectiveness of counting the compresses used before and after the emergency operation versus performing abdominal x-rays before the patient left the surgical center.^2^ Thus, this topic would require a good randomized clinical trial to compare the two approaches: counting the compresses or performing abdominal x-rays in the surgical theater. The study only took into account emergency surgery and calculated the costs of x-rays and the consequences of forgetting the compresses, including the legal costs, but did not find any clinical trials. It is known that emergency surgery has five to nine times greater risk of foreign-body retention in the abdomen, in relation to elective surgery, particularly in cases of major hemorrhage.

It should be borne in mind that the mortality rate following retention of compresses or gauzes may reach 35%.^2^ Since there was no good evidence, the study was based on case series at large emergency services and on questionnaires. In fact, the lack of good-quality evidence ended up impairing these very important cost-effectiveness analyses. Dossett et al.^2^ concluded that performing x-rays in the surgical theater could almost halve the costs, assuming that x-rays would have greater sensitivity and specificity than shown by counting the compresses. In emergency cases, there may be either overestimation or underestimation of the real number of compresses used, but they are easily identified on x-rays because of their radiopacity. Since the clinical decision analysis incorporated observational studies in setting up the clinical decision tree, the results were less convincing that they could have been if there had been a large randomized clinical trial. All this leads to the belief that it would be good sense to spend additional small amounts through performing x-rays than to spend large sums on clinical and legal complications because compresses were forgotten. Nonetheless, to reduce the uncertainties, a simple clinical trial should be conducted. This would provide decisive data for improving medical practice. Since the sample size needed would be very large (more than 10,000 cases in each group), a collaborative study would be needed: hard work but possible. Along the same lines, it is worth remembering that clinical trials have been very important with regard to demonstrating the risks the use of hormone replacement for preventing postmenopausal cardiovascular phenomena, the crucial importance of treatment for high blood pressure to prevent death due to cardiovascular disease among type 2 diabetes cases, the role of alendronate for preventing postmenopausal fracture, the still unbeatable role of magnesium sulfate for preventing and treating eclampsia, etc, etc, etc.^3^^,^^4^^,^^5^

For health professionals and their patients not to capsize in the seas of uncertainty that are full of sharks, clinical trials are like a compass and systematic reviews are like the crow’s nest: the lookout point for scanning the horizon on the ship in which our patients embark, trusting in the captains of Medicine.
